# Respiratory outcomes of onasemnogene abeparvovec treatment for spinal muscular atrophy: national real-world cohort study

**DOI:** 10.1007/s00431-024-05886-9

**Published:** 2024-12-03

**Authors:** Moran Lavie, Mika Rochman, Keren Armoni Domany, Inbal Golan Tripto, Moria Be’er, Omri Besor, Liora Sagi, Sharon Aharoni, Mira Ginsberg, Iris Noyman, Hagit Levine

**Affiliations:** 1 Pediatric Pulmonology Unit, Dana-Dwek Children’s Hospital, Tel Aviv Sourasky Medical Center, Tel Aviv, Israel; 2 Faculty of Medical and Health Sciences, Tel Aviv University, Tel Aviv, Israel; 3 Pediatric Pulmonology Unit, Sylvan Adams Children’s Hospital, E. Wolfson Medical Center, Holon, Israel; 4 Pediatric Pulmonary Unit, Soroka University Medical Center, Ben-Gurion University of the Negev, Beer-Sheba, Israel; 5 Department of Family Medicine, Maccabi Healthcare Services, Tel Aviv, Israel; 6 Pediatric Neurology Unit, Dana-Dwek Children’s Hospital, Tel Aviv Sourasky Medical Center, Tel Aviv, Israel; 7 Neurology Institute, Schneider Children’s Medical Center, Petah-Tikva, Israel; 8 Department of Pediatric Neurology, Wolfson Medical Center, Holon, Israel; 9 Pediatric Neurology Unit, Soroka University Medical Center, Ben-Gurion University of the Negev, Beer-Sheba, Israel; 10 Pulmonary Institute, Schneider Children’s Medical Center, Petah-Tikva, Israel

**Keywords:** Spinal muscular atrophy, Respiratory, Onasemnogene abeparvovec, Nusinersen, Restrictive lung disease

## Abstract

Onasemnogene abeparvovec (OA) is a novel gene replacement therapy for patients with spinal muscular atrophy (SMA). This study provides real-world respiratory data for pediatric SMA patients receiving OA who were assessed before and one year after treatment in a multicenter cohort study conducted from 2019 to 2021. Twenty-five OA-treated SMA patients (23 with type 1 and 2 with type 2; median age at treatment 6.1 months, with a range of 0.36–23 months) were included. Sixteen were treatment-naïve, and nine had received various prior treatments. Two patients died due to respiratory failure during the study period. Of the remaining 23 patients, four were put on non-invasive ventilation (NIV), bringing ventilated patients to a total of ten during the post-treatment year. Three patients required permanent NIV support, while 13 did not require any respiratory support. Ventilation time decreased from 14.3 to 11.1 hours per day, and respiratory hospitalizations decreased by 26% (from 0.76 to 0.57 per life year). Fifteen of the 23 patients maintained full oral nutrition at study closure compared to 20 of the 25 at study initiation. This real-world data analysis demonstrates that OA may improve respiratory outcomes in SMA patients. Importantly, compounding factors, such as age at treatment initiation, treatment combinations, and natural history, may influence the respiratory course, thus highlighting the need for standardized long-term management.

**Table:** 

**What is Known:** *• Respiratory failure is a leading cause of mortality in untreated spinal muscular atrophy type 1 patients.* *• Onasemnogene abeparvovec (OA) improves neurological outcomes, but real-world respiratory data are limited.*	
**What is New:** *• Our real-world analysis suggests OA may improve respiratory outcomes.* *• Age at treatment and treatment combinations may also influence respiratory trajectory.*

**What is Known:**

*• Respiratory failure is a leading cause of mortality in untreated spinal muscular atrophy type 1 patients.*

*• Onasemnogene abeparvovec (OA) improves neurological outcomes, but real-world respiratory data are limited.*

**What is New:**

*• Our real-world analysis suggests OA may improve respiratory outcomes.*

*• Age at treatment and treatment combinations may also influence respiratory trajectory.*

## Introduction

Spinal muscular atrophy (SMA) is a genetic neurological disorder that leads to the degeneration of spinal cord cells, causing progressive muscle weakness and atrophy. It is caused by an autosomal recessive mutation in the survival motor neuron 1 gene (SMN1) [1]. SMA is classified into five types (SMA0-4). SMA type 1 (SMA1) is the most common and severe phenotype, with symptoms appearing before six months of age. Without treatment, most SMA1 patients die before 2 years of age, mainly due to respiratory issues [2, 3].

SMA’s effect on the respiratory system varies based upon the type and degree of muscle function loss [4]. Patients often experience a decline in respiratory muscle function, leading to paradoxical breathing, tachypnea, and an ineffective cough. This results in poor secretion clearance, respiratory infections, and eventually respiratory failure and death in most untreated SMA1 patients [2].

Recent treatments for SMA include nusinersen (Spinraza), onasemnogene abeparvovec (OA: Zolgensma), and risdiplam (Evrysdi), which have significantly improved neurological function and have been shown to alter the course of disease [5–8]. However, there are limited data on long-term respiratory outcomes, highlighting the need for more research on the effects and complications of these treatments [8–11].

To address the scarcity of real-world evidence to complement data from randomized controlled trials, we present this retrospective follow-up study focusing upon the respiratory outcomes of SMA1 and SMA2 pediatric patients who received OA treatment.

## Methods

This multicenter retrospective cohort study included all SMA patients treated with OA at four SMA centers in Israel. These centers specialize in multidisciplinary care and provide comprehensive treatment for most of the SMA patients in the country. The study aimed to understand the respiratory clinical progression in SMA patients after one year of OA treatment.

This study adhered to the principles outlined in the Declaration of Helsinki. Ethical approval was granted by the ethics committees of each participating institution: Tel Aviv Sourasky Medical Center (0873–17-TLV), Schneider Children’s Medical Center (0191–21-RMC), Soroka University Medical Center (031–21-SOR), and Wolfson Medical Center (0179–21-WOMC). Informed consent was waived for this retrospective and anonymized study.

### Subjects

All study participants had a genetically confirmed homozygous deletion or mutation in the SMN1 gene. They showed clinical signs of either SMA1 (symptoms before six months of age) or SMA2 (symptoms between 6 and 18 months of age). We further classified SMA1 into subtypes based upon age at diagnosis: 1A (symptoms within the first two weeks), 1B (symptoms within the first three months), and 1C (symptoms between three and six months) [12]. Newborn screening for SMA is not currently performed in our country.

### OA treatment, procedures, and patient management

Eligibility for gene therapy was determined according to the U.S. Food and Drug Administration (FDA) criteria for OA treatment at the time of the study, which applied to pediatric patients under 2 years of age with spinal muscular atrophy (SMA) and bi-allelic mutations in the SMN1 gene [13]. Each participant received a single intravenous infusion of OA at a standard dose of 1.1 × 10^14^ vector genomes per kilogram of body weight. They also received prednisolone at 1 mg/kg per day, starting 1 day before initiating gene therapy. This prednisolone treatment continued for at least 4 weeks, with dosage adjustments based upon individual needs. Routine blood tests for complete blood count, liver enzymes, and troponin I were performed according to the manufacturer’s protocol. Follow-up protocols varied as did social circumstances affecting adherence due to the participation of different medical centers. All participants were evaluated by the multidisciplinary neuromuscular team of each center with tailored evaluations and interventions based upon their specific needs.

### Data collection

Patients’ data on anthropometric and clinical parameters were collected from medical records, including birth date, sex, number of SMN2 copies, and specific SMA subtypes. Age at diagnosis, age at treatment initiation, and any prior treatments with other SMA-modifying drugs were noted. Respiratory data included the type of assisted ventilation (invasive or non-invasive), categorized as (a) no use, (b) partial use (< 16 h/day), or (c) changed use (≥ 16 h/day for over 14 continuous days without an acute event). Additional respiratory information covered the use of mechanical insufflation-exsufflation (MIE), chest x-ray findings, number of non-elective respiratory hospitalizations, and deaths. The yearly hospitalization rate per patient was calculated by dividing the total hospitalizations by the study duration in days, then adjusting to a 365-day period and averaging across the cohort. Other clinical data included feeding method (oral, nasogastric tube, gastrostomy), weight standard deviation score (SDS), presence of scoliosis, and polysomnography findings. All variables were recorded at treatment initiation (baseline) and repeated 1 year later.

### Descriptive statistics

We reported frequencies and percentages for categorical variables, and means with standard deviations or medians with ranges for continuous variables regarding the outcomes of interest. All analyses were conducted using IBM SPSS Statistics (version 25). Differences between pre- and post-treatment results in each continuous variable were assessed using the paired sample *t* test and Wilcoxon signed-rank test, depending upon their normal distribution. Results were considered statistically significant at a *p* value less than 0.05.

## Results

In this study, 25 SMA patients participated: 23 had SMA1 (11 males, 12 females), and 2 had SMA2 (1 male, 1 female). The median age at diagnosis was 3 months (range, 0.16–17 months). SMN2 copy data were available for 24 patients: 18 had two copies, and 6 had three copies. OA treatment began at a median age of 6.1 months (range, 0.36–23 months). Before receiving OA, 8 patients had received nusinersen, and 1 patient had received risdiplam. Sixteen patients (64%) were treatment-naive. Of the 25 SMA patients, 23 maintained a 1-year follow-up post-OA treatment, while 2 died from respiratory failure. Adherence to follow-up for survivors was 100%. Anthropometric data of the study cohort are listed in Table 1.

**Table 1:** Anthropometric data of the study cohort

	Previously treated	Treatment-naïve	All
N (%)	9 (36%)	16 (64%)	25 (100%)
Median age of diagnosis, months (range)	1.0 (0.2–8)	4.3 (0.16–17)	3 (0.16–17)
Sex			
Male	5 (56%)	7 (44%)	12 (48%)
Female	4 (44%)	9 (56%)	13 (58%)
SMA type			
1a	3 (33.4%)	5 (31.25%)	8 (32%)
1b	4 (44.4%)	8 (50%)	12 (48%)
1c	2 (22.2%)	1 (6.25%)	3 (12%)
2		2 (12.5%)	2 (8%)
Median age at OA treatment, months (range)	16 (3–23)	5 (0.36–20)	6.1 (0.36–23)
Median age at Spinraza/Risdiplam treatment initiation, months (range)	1.69 (0.78–12)		

SMA1 classification-1A—clinical presentation within the first two weeks of infancy, 1B–clinical presentation by age 3 months, 1C–clinical presentation after 3 months and usually before 6 months of age

### Baseline assessment before treatment initiation

Before starting OA treatment, 6 patients (24%) were using noninvasive ventilation: 3 permanently and 3 partially. On average, they used ventilation for 14.3 h per day, and none required invasive ventilation. Twelve patients (48%) used routine daily MIE. Seven patients had been previously hospitalized for acute respiratory events, during which three of them required invasive ventilation. The hospitalization rate was 0.76 per life year. Twenty patients (80%) were fed orally, while 4 (16%) received nutrition through a gastrostomy tube and 1 (4%) through a nasogastric tube. Initial swallowing assessment was based upon clinical findings, and all patients were additionally assessed by videofluoroscopy prior to gastrostomy insertion. The median weight SDS was − 1.51 ± 1.64. Sleep studies were conducted at the discretion of the treating pulmonary physician based upon the presence of symptoms of sleep-disordered breathing, poor weight gain, chest wall deformities, and evidence of hypoventilation (e.g., increased CO_2_ levels). At baseline, seven patients underwent polysomnography that revealed mild-to-moderate obstructive sleep apnea for all, with an apnea–hypopnea index ranging from 1.1 to 7.4 events/h. None exhibited hypoventilation based upon normal CO_2_ monitoring (< 50 EtCO_2_ > 80% of the time).

### Assessment at 1-year post-treatment

NIV was initiated for four additional patients (17%) after 1 year of OA treatment, yielding a total of ten patients (43%) on NIV, with three requiring it permanently and seven partially. Various factors mandated the initiation of ventilatory support, specifically, obstructive sleep apnea (two patients), recurrent respiratory infections with chronic atelectasis (one patient), and prolonged hospitalization leading to respiratory failure (one patient). No patients required chronic invasive ventilation. One of the newly ventilated patients had undergoing prior nusinersen treatment, while the other three were treatment-naïve. All of the patients who used NIV at study closure had been treated with either OA or nusinersen/risdiplam within the first 0.7 to 6 months of life. Patients who were ventilated and had prior treatment with nusinersen/risdiplam began OA between 12 and 23 months of life.

At 1-year post-treatment, the average daily ventilation use was 11.1 h per day. Sixteen of the 23 surviving patients used mechanical insufflation-exsufflation (MIE), with 12 incorporating it into their daily routine and four using it during respiratory exacerbations.

Chest X-rays showed atelectasis in nine patients, pulmonary infiltrates in nine, and a bell-shaped chest in three. Respiratory-related hospitalization frequency decreased from 0.76 to 0.57 per life year during the 1-year follow-up after OA treatment, indicating a 26% decrease. Notably, one of the hospitalized patients was ventilated invasively and subsequently transitioned to NIV during a prolonged stay in intensive care. Four new patients (17%) required gastrostomy during follow-up due to failure to thrive, swallowing difficulties, and confirmed aspirations, totaling eight (35%) on gastrostomy feeding. The median weight SDS for age at 1-year follow-up was − 1.68 (SD ± 1.43), similar to baseline.

During the follow-up year, two patients underwent a repeat sleep study with NIV, yielding normal results. Additionally, three other patients without respiratory support underwent sleep studies, which showed normal results for two patients and an AHI of 1.2 for the third.

At the 1-year follow-up, seven patients were diagnosed with scoliosis, with two displaying it at baseline. Surgical intervention was not deemed necessary for scoliosis in any of these cases at this point, likely due to the young age of these patients.

### Subgroup analysis

We analyzed a subgroup of patients who had not received treatment before the study. Sixteen patients were treatment-naïve, among whom 14 had SMA1 and 2 had SMA2, excluding the latter two from this analysis. Two patients passed away during the first year, leaving 12 treatment-naïve SMA1 patients for analysis. Before starting OA treatment, 3 of these SMA1 patients had used noninvasive ventilation, 2 permanently and 1 partially, averaging 16 h of daily ventilation. Seven patients had used daily MIE. After 1 year of treatment, NIV was initiated for 3 patients, now totaling 6 on NIV, with 2 needing it permanently. None needed chronic invasive ventilation. The mean daily ventilation after 1 year was 11.6 h. MIE was initiated in 4 patients, now totaling 9 using MIE, with 7 incorporating it daily and 2 during respiratory exacerbations.

**Graph 1:**
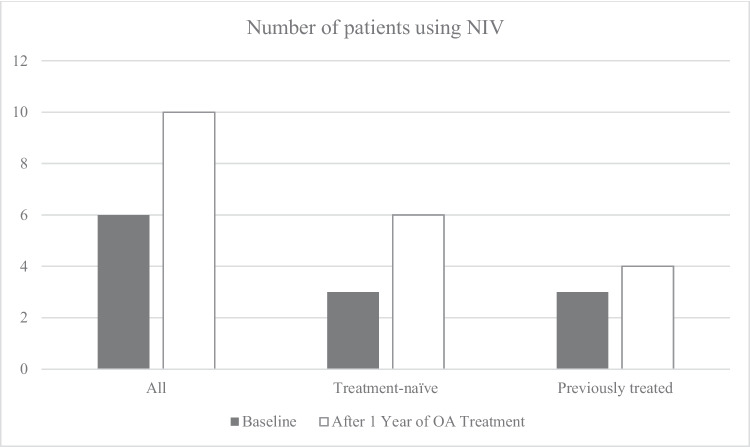
Number of patients using NIV by subgroups: at baseline and after 1 year of treatment

**Graph 2:**
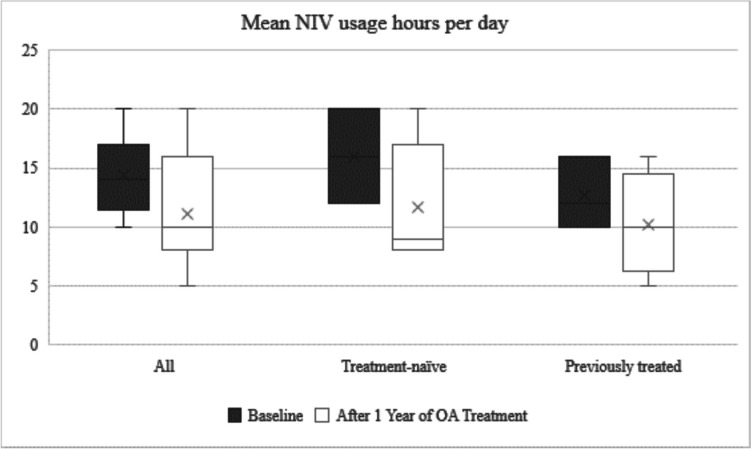
Mean hours of ventilation by subgroups: at baseline and after 1 year of treatment

Before OA treatment, 11 patients were orally fed, 2 through gastrostomy, and 1 via nasogastric tube. The median weight SDS was − 0.925 (SD ± 1.974). Four patients needed a gastrostomy during follow-up, now totaling 6 on gastrostomy feeding. The median weight SDS at one-year follow-up was − 1.405 (SD ± 1.41). Respiratory-related hospitalizations decreased from 2.1 to 0.75 per life year after 1 year of OA treatment, a decrease of 64%. Respiratory data are presented in Table 2 and Graphs 1 and 2.

**Table 2:** Respiratory outcomes

	Baseline	After 1 year of OA
*All*	(*n* = *25*)	(*n* = *23*)*
Use of NIV—Yes n (%)	6 (24%)	10 (43%)
Mean ventilation hours/day	14.3	11.1
Use of MIE		
Daily	12 (48%)	12 (52.2%)
During exacerbation	0	4 (17.4%)
Respiratory hospitalization/life year	0.76	0.56
*Treatment-naïve subgroup*	(*n* = *16*)	(*n* = *14*)
Use of NIV—Yes *n* (%)	3 (18.8%)	6 (43%)
Mean ventilation hours/day	16	11.6
Use of MIE		
Daily	7 (44%)	7 (50%)
During exacerbation	0	2 (14%)

^*^Excluding two patients who died

### Treatment complications and mortality

No respiratory complications or medication-related side effects were reported in any of the patients during or shortly after OA treatment. Two patients died of respiratory failure during the year post-treatment.

## Discussion

This multicenter cohort study examined the effects of OA treatment on respiratory outcomes in SMA patients in real-world settings. Our results showed that OA treatment may influence the respiratory course of SMA patients compared to historical cohorts, but that it does not eliminate the need for ongoing respiratory monitoring and support.

One year after OA treatment, the number of non-invasively ventilated patients increased to 10 out of 23 (43%) patients. These findings match those of Stettner et al. and Panagiotou et al., highlighting the continued need for respiratory support after OA treatment [14, 15]. Similarly, in the STR1VE and STR1VE-EU phase 3 trials, and the Five-Year Follow-up Extension of the Phase 1 START trial, 32%, 61%, and 40% of patients, respectively, still needed daily ventilatory support (invasive or non-invasive) [5–7]. Notably, even though the drug was given earlier (3.7 and 4.1 months, respectively) in these trials compared to our study, the need for respiratory support persisted [5–7]. Therefore, a possible explanation for the increased need for NIV in our cohort may be the number of non-naïve patients receiving OA treatment at a later age, while another possibility may be a more severe phenotype.

Our findings show a positive outcome in ventilated patients, with reduced average daily ventilation hours, aligning with clinical trials that reported significant improvements or cessation of ventilator support after OA treatment [16–18]. Sixteen of our participants were treatment-naive, and although daily ventilator use improved, none were fully weaned off ventilation. Previous reports mainly focused upon SMA1 patients who received intrathecal nusinersen before OA treatment [16], possibly affecting the outcomes. For example, Al Naimi et al. found that 10 out of 11 patients stopped NIV over a 2-year follow-up, with all having received nusinersen before OA treatment. These findings suggest that prior nusinersen might have improved the respiratory state before OA treatment, or there could be a synergistic effect between the two drugs, leading to better respiratory outcomes.

Another encouraging result in our study was a reduction in the rate of respiratory-related hospitalizations. Our findings showed 0.57 respiratory hospitalizations per year in the whole cohort and 0.75 per year in treatment-naive SMA1 patients, compared to the 1.4 rate reported by Al-Zaidy et al. [17]. This reduction in hospitalizations is significant for patients and their families by helping to maintain the patient's quality of life at home, which is pivotal to their overall physical and mental well-being. Hospitalization can hinder the progress of neurological, respiratory, and nutritional health and expose vulnerable children to infection risks and additional complications. Moreover, reducing hospitalization days is cost-effective for both the healthcare system and the patient’s caregivers.

Feeding and swallowing abilities are crucial for maintaining respiratory health in SMA patients. Before treatment, 20 out of our 25 (80%) patients had full oral nutrition. One year after treatment, 15 (65%) patients maintained full oral nutrition. These results align with a post-hoc analysis of data from the START, STR1VE-US, and STR1VE-EU studies, which showed that 85% (55/65) of patients had full oral nutrition pre-treatment and 75% (49/65) maintained it at the last evaluated time point [5–7, 19]. Notably, only 6 out of 12 treatment-naive SMA1 patients in our study could be maintained solely on oral feeding one year after starting treatment.

Throughout our study, we identified additional factors related to respiratory progression in pediatric SMA patients, such as sleep-disordered breathing, abnormal lung imaging findings, and scoliosis. Although these issues are discussed in the literature [20, 21], our small sample size limits our ability to draw definitive conclusions. Combining our results with those of other studies [20, 21], however, provides insights into key factors affecting the respiratory impact of OA treatment in SMA and highlights important issues that need to be considered. First, the age at treatment initiation is crucial. Given the progressive nature of muscle weakness in SMA, younger and less symptomatic children are likely to experience a more significant impact from the treatment. Second, the differences between treatment-naive patients and those previously treated with an SMA-modifying drug, observed by us and others, suggest possible synergy between treatments and combined positive effects on respiratory outcomes. Lastly, the lack of standardized guidelines for respiratory care in children undergoing OA treatment, especially regarding ventilator weaning and reducing supportive care, leads to varied practices across medical centers. This underscores the importance of collecting and analyzing data to develop evidence-based guidelines.

Our study’s limitations stem from the small patient cohort, reflecting the rare nature of the disease. The small number of patients in the study precluded the achievement of statistically significant results. Additionally, the 1-year observation period might not fully capture the long-term trajectory and prognosis of patients. Moreover, the absence of standardized respiratory care guidelines for children undergoing OA treatment may have led to variations in care across the four participating centers.

In conclusion, OA shows promising potential in positively influencing the respiratory course of SMA patients. However, it is challenging to differentiate between the positive effects of treatment, including the timing of treatment initiation and previous exposure to other disease-modifying therapies, and those attributed to natural changes as patients grow. Our real-world findings emphasize the need for comprehensive, standardized, long-term respiratory management of SMA, despite advancements provided by gene therapy. Further research to deepen our understanding of factors influencing the clinical status and prognosis of pediatric SMA patients undergoing gene replacement therapy is warranted.

## Data Availability

No datasets were generated or analyzed during the current study.
